# Pharmacokinetics and safety of dolutegravir in term neonates exposed to HIV-1 (IMPAACT 2023): a phase 1, open-label, multicentre study

**DOI:** 10.1016/S2352-3018(26)00226-2

**Published:** 2026-10-21

**Authors:** Jeremiah D Momper, Jiajia Wang, Sarah Bradford, Iris Mustich, Benjamin Johnston, Kyle Whitson, Madison O’Brien, Evan Goldberg, Dwight E Yin, Ellen Townley, Kelly Colsh, Tara DeYampert, Ceora Beijer-Spell, Orasri Wittawatmongkol, Mariam Aziz, Megeshinee Naidoo, Suchada Ruenglerdpong, Adrie Bekker, Jaime G Deville, Maria Leticia Santos Cruz, Sisinyana Ruth Mathiba, Ann M Buchanan, Michael McKenna, Rashmi Mehta, Hardik Chandasana, Edward P Acosta, Brookie M Best, Mark Mirochnick, Kathleen M Powis, David E Shapiro, Diana F Clarke

**Affiliations:** Skaggs School of Pharmacy and Pharmaceutical Sciences, University of California San Diego, La Jolla, CA, USA (Prof J D Momper PhD, Prof B M Best PharmD); **Center for Biostatistics in AIDS Research, Harvard T H Chan School of Public Health, Boston, MA, USA** (J Wang MS, E Goldberg MS, D E Shapiro PhD); **FHI 360, Durham, NC, USA** (S Bradford MPH, I Mustich MPH); **Frontier Science Foundation, Amherst, NY, USA** (B Johnston BS, K Whitson MA, M O’Brien MA); **Division of AIDS, National Institute of Allergy and Infectious Diseases, National Institutes of Health, Rockville, MD, USA** (D E Yin MD, E Townley FNP, K Colsh PharmD); **Eunice Kennedy Shriver National Institute of Child Health and Human Development, National Institutes of Health, Bethesda, MD, USA** (T DeYampert MD); **ACTG/IMPAACT Laboratory Center at UCLA, Los Angeles, CA, USA** (C Beijer-Spell BS); **Division of Pediatric Infectious Diseases, Department of Pediatrics, Faculty of Medicine, Siriraj Hospital, Mahidol University, Bangkok, Thailand** (O Wittawatmongkol MD); **Rush University Medical Center and Rush Medical College, Chicago, IL, USA** (M Aziz MD); **CAPRISA and Department of Paediatrics and Child Health, University of KwaZulu-Natal, Durban, South Africa** (M Naidoo MBChB); **Chiangrai Prachanukroh Hospital, Chiang Rai, Thailand** (S Ruenglerdpong MD); **Family Centre for Research with Ubuntu, Department of Paediatrics and Child Health, Stellenbosch University, Cape Town, South Africa** (Prof A Bekker PhD); **David Geffen School of Medicine at UCLA and UCLA Mattel Children’s Hospital, Los Angeles, CA, USA** (Prof J G Deville MD); **Infectious Diseases Department, Hospital Federal dos Servidores do Estado, Rio de Janeiro, Brazil** (M L Santos Cruz MD); **Perinatal HIV Research Unit, Chris Hani Baragwanath Academic Hospital, University of the Witwatersrand, Johannesburg, South Africa** (S R Mathiba MBChB); **ViiV Healthcare, Durham, NC, USA** (A M Buchanan MD); **GSK, London, UK** (M McKenna MBChB); **Clinical Pharmacology Modeling and Simulation, GSK, Collegeville, PA, USA** (R Mehta PhD, H Chandasana PhD); **Department of Pediatrics, Division of Pediatric Infectious Diseases, Clinical Pharmacology Laboratory and Pharmacometrics Core, University of Alabama at Birmingham School of Medicine, Birmingham, AL, USA** (Prof E P Acosta PharmD); **Department of Pediatrics, Boston University Chobanian & Avedisian School of Medicine and Boston Medical Center, Boston, MA, USA** (Prof M Mirochnick MD, D F Clarke PharmD); **Massachusetts General Hospital and Harvard Medical School, Boston, MA, USA** (K M Powis MD)

## Abstract

**Background:**

Safe and effective antiretroviral options are needed for neonates exposed to HIV-1, but data supporting dolutegravir use from birth are scarce. The aim of this study was to evaluate dolutegravir pharmacokinetics, safety, and tolerability in term neonates, and to identify an appropriate dosing regimen.

**Methods:**

IMPAACT 2023 was a phase 1, open-label, multicentre study conducted at 15 IMPAACT sites in South Africa, Thailand, and the USA from Oct 5, 2022, to May 22, 2025. Full-term neonates weighing at least 2 kg born to women living with HIV-1 were enrolled between birth and 5 days of life. In cohort 1, infants received two single dolutegravir doses using liquid suspension or 5 mg dispersible tablets. In cohort 2, infants received dolutegravir 5 mg every 48 h until day 14 of life, then every 24 h up to week 4–6 depending on duration of local standard-of-care antiretroviral prophylaxis. In both cohorts, participants were stratified by antepartum maternal dolutegravir exposure. Intensive pharmacokinetic sampling was done around each of the two single doses in cohort 1 and at 7 days post initial dose and at week 4 of life in cohort 2. Safety and tolerability were assessed up to week 16. Primary pharmacokinetic outcomes were the last observed concentration of dolutegravir after each dose and area under the observed single-dose concentration–time curve in cohort 1, and concentration of dolutegravir at the end of the dosing interval (C_trough_) and area under the concentration–time curve over one dosing interval (AUC_0–τ_) in cohort 2. Pharmacokinetic parameters were estimated by non-compartmental analysis in infants who received the dolutegravir dose under evaluation and had evaluable pharmacokinetic data from intensive sampling. Primary safety outcomes were study drug-related safety failure and safety failure from initial dosing up to 2 weeks after permanent study drug discontinuation. Safety outcomes were assessed in all infants who received at least one dose of dolutegravir and were summarised descriptively. The study is registered with ClinicalTrials.gov, NCT05406583, and is complete.

**Findings:**

Of 73 screened mother–infant pairs, 48 were enrolled between Oct 5, 2022, and Jan 21, 2025. All 48 infants started the study drug and were safety-evaluable; 41 were pharmacokinetic-evaluable. In cohort 1, concentration–time profiles showed prolonged elimination and wide interindividual variability, with substantial overlap between neonates with and without antepartum maternal dolutegravir exposure. In cohort 2, geometric mean C_trough_ and AUC_0–τ_ at the day 7 post initial dose visit were within target ranges. At week 4 of life, geometric mean AUC_0–τ_ remained within target range, whereas C_trough_ exceeded the target range but remained acceptable. No infants in either cohort had a study drug-related safety failure during the primary safety window. Safety failure occurred in zero of 18 infants in cohort 1 and four of 30 infants in cohort 2. Two of 48 infants had grade 1–2 adverse events that were considered related to dolutegravir.

**Interpretation:**

These findings support dolutegravir as an option from birth for presumptive therapy or early treatment in term infants weighing at least 2 kg.

**Funding:**

National Institutes of Health, ViiV Healthcare, and GlaxoSmithKline.

## Introduction

Despite major advances in HIV prevention and treatment, more than 1 million neonates worldwide are born to women living with HIV annually^1^ and require safe and effective antiretroviral therapy (ART) regimens from birth for presumptive therapy or early treatment. US and WHO guidelines recommend three-drug ART regimens as presumptive therapy or enhanced prophylaxis for infants at high risk of HIV acquisition.^2,3^ Early initiation of ART in neonates living with HIV offers important clinical advantages, including limiting early viral replication, reducing seeding of long-lived viral reservoirs, preserving immune function, and potentially enabling future treatment interruptions or novel remission strategies.^4–6^ Neonatal pharmacokinetic and safety data are available for zidovudine, lamivudine, nevirapine, and raltegravir, components of contemporary neonatal regimens. However, toxicity, formulation limitations, dosing complexity, and lower resistance barriers continue to constrain practical options in the immediate neonatal period.^7–9^

Second-generation integrase strand transfer inhibitor (INSTI)-based regimens are preferred for initial therapy in infants, children, and adults because of their potency, high genetic barrier to resistance, and favourable safety profile.^10,11^ Raltegravir, a first-generation INSTI, is approved for use in neonates, but its lower resistance barrier and dosing complexities limit its clinical utility. Dolutegravir, a second-generation INSTI, is therefore an attractive alternative; however, its pharmacokinetics and safety in the immediate neonatal period have not been fully characterised. At study initiation, approvals for dolutegravir dispersible tablets began at age 4 weeks and weight of at least 3 kg, leaving a critical gap during the first weeks of life. In 2025, the PETITE-DTG study reported appropriate pharmacokinetic exposures and acceptable safety with dolutegravir dispersible tablets and oral films in term neonates born to women receiving long-term dolutegravir-based therapy.^12^ WHO has identified development of neonatal dolutegravir dosing as a global research priority, given increasing availability of generic dolutegravir dispersible tablets and widespread use of dolutegravir in pregnant women with HIV.^13^ Consequently, many neonates have residual transplacental dolutegravir exposure at birth, raising the question of whether dolutegravir-exposed and dolutegravir-naive infants require different dosing. To address this gap, IMPAACT 2023 was designed to evaluate the pharmacokinetics, safety, and tolerability of dolutegravir administered with standard background neonatal ART regimens in term neonates born to women living with HIV-1 from birth until 4–6 weeks of life, and to identify an appropriate dolutegravir dosing regimen.

## Methods

### Study design

IMPAACT 2023 was a phase 1, multisite, open-label, non-randomised study with two sequential cohorts stratified by maternal dolutegravir use. Infants were defined as dolutegravir-naive or dolutegravir-exposed according to maternal dolutegravir exposure: dolutegravir-naive infants had no maternal dolutegravir exposure during the 2 weeks before birth, whereas dolutegravir-exposed infants were born to mothers who received at least one dolutegravir dose within 72 h before delivery. The study was conducted at 15 sites affiliated to hospitals and universities in the International Maternal Pediatric Adolescent AIDS Clinical Trials (IMPAACT) Network in South Africa, Thailand, and the USA from Oct 5, 2022, to May 22, 2025. The study was approved by the Advarra single institutional review board for US participating sites (protocol number Pro00058911). International sites obtained local ethics and regulatory approvals (appendix p 16). An independent IMPAACT Study Monitoring Committee periodically reviewed accumulating safety, tolerability, and pharmacokinetic data and provided recommendations regarding continuation, modification, or termination of enrolment and dosing. A protocol overview is provided in the appendix (p 2), and the full protocol is available on the IMPAACT website.^14^ The study is registered with ClinicalTrials.gov, NCT05406583, and is complete.

Community representatives were involved in protocol development and study implementation through the IMPAACT Community Advisory Board. Community representatives served on the IMPAACT 2023 protocol team and provided community and potential-participant perspectives during protocol development and study implementation. IMPAACT community engagement and publication processes also supported community input into study materials, review, and dissemination of study results.

### Participants

Singleton, generally healthy term infants (born at ≥37 weeks’ gestation) with birthweight of at least 2 kg, who were born to women living with HIV-1, were screened. To be eligible, infants had to have received at least one dose of standard-of-care antiretroviral prophylaxis before study entry. Infants were excluded if they had a positive HIV-1 nucleic acid test at screening or entry, known maternal–fetal blood group incompatibility, previous exchange transfusion, hyperbilirubinaemia requiring exchange transfusion, or receipt of (or anticipated exposure to) disallowed medications either directly or through breastmilk. Biological sex was recorded from the infant’s clinical record at enrolment. Race and ethnicity were collected at enrolment, defined according to US National Institutes of Health (NIH)–Office of Management and Budget categories. All maternal participants provided written informed consent for themselves and their infant before study entry.

Mothers and infants were enrolled as pairs when infants were aged 0–5 days; mothers did not receive study drug and exited the study after the entry visit. Infants received dolutegravir in addition to standard-of-care antiretroviral prophylaxis according to local practice and were followed up until a week 16 study visit (days 112–140 of life). Standard-of-care antiretroviral prophylaxis comprised single-drug or multidrug regimens that included zidovudine, nevirapine, or lamivudine. The study used an adaptive, model-informed design with two sequential cohorts termed cohort 1 and cohort 2. The initial dose-finding target was 36 evaluable infants: six in each of two strata in cohort 1, and 12 in each of two cohort 2 strata. Opening of the conditional cohort 1 stratum 1C added a target of six evaluable infants. Enrolment of up to 108 mother–infant pairs was permitted to replace unevaluable infants and to allow evaluation of a modified dose or formulation if prespecified pharmacokinetic or safety criteria were not met.

### Procedures

In cohort 1, two single doses of dolutegravir were given with standard-of-care ART used for prophylaxis in the first 15 days of life. Infants were stratified by antepartum maternal dolutegravir exposure before birth and enrolled into one of three strata. Infants in stratum 1A (dolutegravir-naive) received two single 0·5 mg/kg doses of investigational dolutegravir liquid suspension in a medium-chain triglyceride vehicle, with the first dose at 0–5 days of life and the second 7–10 days later. Infants in stratum 1B (dolutegravir-exposed) received the same dolutegravir liquid suspension regimen, with the first dose at 2–5 days of life and the second 7–10 days later. After enrolment of six evaluable infants per stratum, accrual was prospectively paused for interim analysis. Safety and pharmacokinetic data were reviewed by the protocol team and the independent study monitoring committee, according to prespecified criteria. Progression to the dispersible tablet formulation and selection of subsequent dosing required acceptable short-term safety, defined as no dose-limiting or unexpected grade 3 or higher toxicities attributable to dolutegravir, and pharmacokinetic modelling and simulation supporting the selected formulation and dosing. Because these criteria were met, stratum 1C (dolutegravir-naive) was opened to enrolment; infants in this stratum received two single doses of one 5 mg dispersible tablet for oral suspension, with the first dose administered at 0–5 days of life and the second 7–10 days later. Pharmacokinetic sampling around the first dose was done before the dose and at 1–2 h, 4–8 h, 11–13 h, 22–26 h, and 48–72 h post dose; around the second dose, sampling was done before the dose and at 1–2 h and 22–26 h post dose. Cohort 1 results informed the population pharmacokinetic modelling and simulation used to select the chronic dolutegravir dosing regimen and formulation evaluated in cohort 2.^15^

Enrolment into cohort 2 proceeded after confirmation by the study monitoring committee that cohort 1 safety and pharmacokinetic data supported progression to cohort 2. Infants were enrolled into one of two strata: stratum 2A (dolutegravir-naive) and stratum 2B (dolutegravir-exposed). In both strata, dolutegravir 5 mg dispersible tablets were administered every 48 h until day 14 of life, followed by once-daily dosing up to week 4–6 depending on duration of local standard-of-care antiretroviral prophylaxis. Infants received their first dolutegravir dose at 0–5 days of life. Pharmacokinetic sampling was done 7 days after dolutegravir initiation and at week 4 of life. The day 7 visit was prespecified to characterise early repeated-dose exposure under the cohort 2 regimen selected using cohort 1 pharmacokinetic data and modelling, and week 4 sampling evaluated exposure after further postnatal maturation. For infants still receiving doses every 48 h at the day 7 visit, samples were collected before the dose and at 1–2 h, 6–10 h, 22–26 h, and 48 h post dose, with the 48 h sample obtained before administration of the next dose. For infants who had transitioned to once-daily dosing, samples were collected before the dose and at 1–2 h, 6–10 h, and 22–26 h post dose.

The safety and tolerability of dolutegravir administered with standard antiretroviral prophylaxis were evaluated up to week 16. Tolerability was assessed from first dose until study drug discontinuation and included caregiver report on ease of swallowing the dolutegravir dose and problems the infant experienced when taking dolutegravir. Adverse events were systematically assessed at each study visit and graded with the Division of AIDS Table for Grading the Severity of Adult and Pediatric Adverse Events, corrected version 2.1,^16^ with the exceptions of axillary fever and creatinine, which used protocol-specific grading. Attribution to study drug was assigned by site investigators.

Prespecified protocol criteria were used to guide study drug interruption, modification, or permanent discontinuation at the individual participant level in both cohorts. Study drug was to be temporarily withheld or permanently discontinued for any infant who developed grade 2 alanine aminotransferase (ALT) elevation, grade 2 rash with elevated ALT, or a grade 3 or higher adverse event, had total bilirubin greater than 16·0 mg/dL, or underwent exchange transfusion. If HIV-1 infection was confirmed, dolutegravir was to be permanently discontinued.

Dolutegravir plasma concentrations were quantified by use of liquid chromatography with tandem mass spectrometry at the IMPAACT Pharmacology Support Laboratory at the University of Alabama at Birmingham (Birmingham, AL, USA), as previously described.^17^ Dolutegravir was extracted from 20 μL of plasma and the assay had lower and upper limits of quantification of 5 ng/mL and 10 000 ng/mL, respectively.

Non-compartmental pharmacokinetic analysis used Phoenix WinNonlin version 8.6 (Certara, Princeton, NJ, USA). Dolutegravir pre-dose concentrations, maximum concentrations (C_max_), and corresponding times were observed directly. C_last_ was the last observed concentration after each cohort 1 single dose, and C_trough_ was the concentration at the end of the dosing interval in cohort 2. The area under the observed single-dose concentration–time curve (AUC) for cohort 1 and the area under the concentration–time curve over one dosing interval (AUC_0–τ_) for cohort 2 were estimated with the linear-up–log-down trapezoidal method. For cohort 2 participants without a collected 48 h trough sample, C48 was interpolated from preceding terminal-phase concentrations. The protocol-defined cohort 2 targets were selected to approximate established adult and paediatric dolutegravir exposures while limiting overexposure. These were population geometric mean benchmarks rather than validated individual therapeutic ranges. The target population geometric mean C_trough_ was 0·995 μg/mL (acceptable range 0·697–2·26 μg/mL), the target AUC_0–τ_ was 46·0 μg·h/mL (37·0–134 μg·h/mL), and the upper limit for C_max_ was 18·4 μg/mL. The lower bounds for C_trough_ and AUC_0–τ_ were 70% and 80% of their respective target geometric means. The C_max_ limit was set at five times the typical adult geometric mean C_max_ because of the theoretical concern for bilirubin displacement at supratherapeutic dolutegravir concentrations.

### Outcomes

The primary objectives were to evaluate dolutegravir pharmacokinetics, safety, and tolerability during the first 4–6 weeks of life and to propose an appropriate dolutegravir dosing regimen for term neonates. The two primary safety outcomes were the proportions of infants experiencing study drug-related safety failure or safety failure from initial study drug dosing up to 2 weeks after permanent study drug discontinuation. An infant was classified as experiencing a study drug-related safety failure if any of the following occurred and was assessed as related to dolutegravir: a grade 3 or 4 adverse event, death, a life-threatening adverse event, or an adverse event leading to premature permanent study drug discontinuation. An infant was classified as having a safety failure if any grade 3 or 4 adverse event or death occurred. The two secondary safety outcomes were the proportions of infants experiencing study drug-related safety failure or safety failure up to week 16. The primary tolerability outcome was inability to tolerate study drug, as defined in the protocol. In cohort 1, in which isolated single doses were evaluated, the primary dolutegravir pharmacokinetic outcomes were C_last_ and AUC. In cohort 2, in which repeated dosing was evaluated, the primary pharmacokinetic outcomes were C_trough_ and AUC_0–τ_.

### Statistical analysis

Target sample size was based on the precision required for the population pharmacokinetic modelling and simulation used for dose selection, specifically 80% power for a 95% CI within 60–140% of the geometric mean estimate of neonatal dolutegravir oral clearance. For the primary safety outcomes, the precision of estimation (CI width) provided by the target sample size was calculated for a range of potential safety failure proportions.

The final demographic, safety, and tolerability analyses were conducted in the all-treated population (all infants who received at least one dose of dolutegravir). The pharmacokinetic analysis was conducted in the pharmacokinetic-evaluable population (infants who received the dolutegravir dose under evaluation and had evaluable pharmacokinetic data from intensive sampling).

The main safety analyses were based on observed data through each infant’s last completed safety assessment. Point estimates and exact binomial (Clopper–Pearson) 90% CIs were calculated for the proportions of infants who had at least one safety or tolerability outcome. Sensitivity and supplementary analyses addressing the potential impact of missing data due to premature study discontinuation, alternative tolerability definitions, and missing 48-h pharmacokinetic samples are described in the appendix (pp 3–5). Statistical analyses were performed using SAS version 9.4 software.

### Role of the funding source

The US NIH, ViiV Healthcare, and GlaxoSmithKline (GSK) provided funding for the study, and ViiV Healthcare and GSK provided the study drug. Study design and data interpretation were undertaken by the protocol team, which included representatives of the NIH, ViiV Healthcare, and GSK. Data collection and analysis were undertaken by the IMPAACT Network. All authors, including representatives of the NIH, ViiV Healthcare, and GSK, critically reviewed the manuscript, approved the final version, and had final responsibility for the decision to submit for publication.

## Results

Between Oct 5, 2022, and Jan 21, 2025, 73 mother–infant pairs were screened, and 48 were enrolled (18 in cohort 1 and 30 in cohort 2; table 1). The final study follow-up visit occurred on May 22, 2025. Across both cohorts, concomitant neonatal antiretroviral prophylaxis included zidovudine in 44 (92%) of 48 infants, nevirapine in 24 (50%), and lamivudine in eight (17%).

All 48 infants started study drug and 44 completed the regimen (all 18 in cohort 1 and 26 of 30 in cohort 2; figure 1). 41 infants were both safety-evaluable and pharmacokinetic-evaluable for the interim dose-finding analyses: 18 in cohort 1 (12 dolutegravir-naive and six dolutegravir-exposed) and 23 in cohort 2 (11 dolutegravir-naive and 12 dolutegravir-exposed). Two additional infants in cohort 2 (one dolutegravir-naive and one dolutegravir-exposed) were safety-evaluable but not pharmacokinetic-evaluable for the interim dose-finding analyses. All infants were evaluable for the final safety analyses. 43 infants (all 18 in cohort 1 and 25 of 30 in cohort 2) completed study follow-up; two infants were lost to follow-up and three were withdrawn from the study (withdrawal of consent by a parent or guardian). No infants were diagnosed with HIV infection during conduct of the study.

In cohort 1, six participants were enrolled into each stratum: 1A (dolutegravir-naive, 0·5 mg/kg liquid suspension), 1B (dolutegravir-exposed, 0·5 mg/kg liquid suspension), and 1C (dolutegravir-naive, 5 mg dispersible tablet). Among stratum 1B participants, pre-dose dolutegravir concentrations attributable to in utero exposure ranged from 0·025 μg/mL to 2·050 μg/mL, with samples collected 44·5–131·7 h after birth. After the first dose, geometric mean C_last_ was 0·212 μg/mL, 0·488 μg/mL, and 1·11 μg/mL, and geometric mean AUC was 23·3 μg·h/mL, 63·5 μg·h/mL, and 113 μg·h/mL in strata 1A, 1B, and 1C, respectively (six infants per stratum). After the second dose, geometric mean C_last_ was 0·265 μg/mL, 0·636 μg/mL, and 1·98 μg/mL, and geometric mean AUC was 8·77 μg·h/mL, 19·9 μg·h/mL, and 52·9 μg·h/mL in the corresponding strata. Concentration–time profiles showed substantial interindividual variability and overlap across strata (figure 2). As previously described, a population pharmacokinetic model was developed with cohort 1 data and used to simulate various chronic dosing regimens with the dolutegravir 5 mg dispersible tablet formulation.^15^ Based on these simulations, a dosing regimen of dolutegravir 5 mg dispersible tablet every other day until day 14 of life, followed by once daily dosing, was selected for evaluation in cohort 2.

Cohort 2 non-compartmental pharmacokinetic analyses were based on the 23 evaluable infants (11 dolutegravir-naive in stratum 2A and 12 dolutegravir-exposed in stratum 2B). Because the visit could occur 7–10 days after dolutegravir initiation, two stratum 2A infants who initiated dolutegravir on day 5 of life had reached the day-14 dosing transition and were receiving doses every 24 h at the visit, whereas the remaining 21 infants were receiving doses every 48 h. Visual inspection of concentration–time profiles suggested some differences in median concentrations between dolutegravir-exposed and dolutegravir-naive infants (figure 3); however, there was substantial overlap, marked interindividual variability, and small sample sizes within strata. Therefore, pharmacokinetic results were pooled.

Among the 21 infants who were still receiving doses every 48 h at the 7 days post initial dose visit, geometric mean C_trough_, AUC_0–τ_, and C_max_ were within the target exposure ranges (table 2). The results were similar in a sensitivity analysis excluding the five participants who had their last 48-hourly dose (first 24-hourly dose) at 7 days post initial dose and did not have a 48-h sample collected (appendix pp 3–4). At the week 4 visit, geometric mean C_trough_ was above the target range, whereas AUC_0–τ_ and C_max_ were within the target ranges (table 2).

In safety analyses, 40 (83%) of 48 infants had at least one adverse event. No adverse events were life-threatening, fatal, or led to study drug interruption or discontinuation. Site investigators could not rule out relationships to study intervention for two adverse events (one grade 2 oral candidiasis and one grade 1 neonatal rash); neither was serious. One participant had a serious adverse event of acute respiratory failure due to bronchiolitis with hypoxia, which was considered unrelated to study drug.

In cohort 1, up to week 16, 14 (78%) of 18 infants had one or more adverse events, none of which met the definition of safety failure (grade 3 or 4 adverse event or death; estimate 0%, 90% CI 0–15). The most frequent adverse event was decreased haemoglobin, occurring in two infants up to 2 weeks after dolutegravir discontinuation and in ten infants up to week 16. No adverse events were assessed as related to study intervention or classified as serious adverse events.

In cohort 2, most adverse events occurred during dosing or in the first 2 weeks after dolutegravir discontinuation. Up to week 16, 26 (87%) of 30 infants had one or more adverse events. The most frequent adverse event was decreased haemoglobin, which occurred in 15 infants. Seven infants (23%, 90% CI 12–39) had at least one safety failure up to week 16; four (13%, 5–28) had a safety failure within 2 weeks after dolutegravir discontinuation, and an additional three experienced a safety failure before the week 16 visit. Of these safety failure adverse events, two were grade 4 (increased blood potassium, possibly attributable to sample haemolysis without other signs of renal toxicity; and decreased neutrophil count) and ten were grade 3 (three increased total bilirubin; one each of decreased neutrophil count, neutropenia, decreased white blood cell count, decreased haemoglobin, acute respiratory failure, bronchiolitis, and hypoxia). None of the grade 3 or 4 adverse events were assessed as related to study intervention and none of the infants received phototherapy for treatment of hyperbilirubinaemia. Reporting of adverse events up to week 16 was similar between dolutegravir-naive and dolutegravir-exposed infants.

Dolutegravir was well tolerated in both cohorts, with no reports of inability to tolerate study drug.

## Discussion

Pharmacokinetic and safety data support a neonatal dolutegravir regimen of one 5 mg dispersible tablet every 48 h during the first 2 weeks of life, followed by once-daily dosing until 4 weeks of age for term neonates with birthweight of 2 kg or greater, regardless of in utero dolutegravir exposure. Dolutegravir exposure with the neonatal regimen was within or above the prespecified target range. Dolutegravir disposition was similar in infants with and without in utero dolutegravir exposure, and the safety profile was consistent with current dolutegravir labelling for infants and children.

Cohort 1 provided single-dose pharmacokinetic data showing prolonged elimination and substantial interindividual variability, consistent with known variability in neonatal maturation of UGT1A1 activity and of gut function.^18^ Infants with maternal dolutegravir exposure had measurable pre-dose concentrations that contributed to higher early total concentrations in some participants, although concentration–time profiles overlapped substantially with those of dolutegravir-naive infants. Although traditional phase 1 pharmacokinetic and safety studies are difficult to conduct in neonates, population pharmacokinetic modelling and simulation can inform selection of initial dosing regimens for evaluation in this vulnerable population, thereby improving study safety. An adaptive design incorporating population pharmacokinetic modelling and simulation was used to determine an appropriate chronic dosing regimen for term infants weighing at least 2 kg at birth. In cohort 2, chronic dosing led to geometric mean C_trough_, AUC_0–τ_, and C_max_ values within target ranges at 7 days post initial dose. At week 4, exposures remained within or above target, with C_trough_ exceeding the target range but AUC_0–τ_ and C_max_ remaining within acceptable bounds. Pharmacokinetic exposure profiles were similar between dolutegravir-exposed and dolutegravir-naive infants, supporting the use of a unified dosing strategy regardless of maternal dolutegravir use.

Across both cohorts, dolutegravir was well tolerated and the safety profile was consistent with current dolutegravir labelling for infants and children. Only two infants had study drug-related adverse events, both of which were mild. No adverse event led to drug discontinuation, and no infant was unable to tolerate the dispersible tablet formulation. The absence of drug-related safety failures supports the acceptability of dolutegravir during the neonatal period.

These findings expand previous neonatal pharmacokinetic studies of integrase inhibitors. In IMPAACT P1026s, washout pharmacokinetics of dolutegravir were evaluated in infants born to mothers with HIV-1 receiving dolutegravir.^19^ Dolutegravir readily crosses the placenta, and elimination after birth had a median half-life of 32·8 h, several fold longer than that in older children and adults.

Dolutegravir is primarily metabolised by UGT1A1, which also mediates bilirubin glucuronidation. Low UGT activity at birth, with maturation over the first weeks to months of life, is likely to have contributed to the interindividual variability in dolutegravir pharmacokinetics observed in this neonatal population. Other contributors might have included birthweight, gestational age, feeding type, race or ethnicity, antepartum maternal dolutegravir exposure, concomitant antiretrovirals such as nevirapine, and genetic variation in UGT1A1. Because dolutegravir binds albumin with higher affinity than bilirubin, markedly supratherapeutic concentrations could displace unconjugated bilirubin from albumin. However, this interaction is unlikely to be clinically significant at typical therapeutic concentrations,^20,21^ underscoring the importance of neonatal dosing regimens that avoid excessive drug accumulation.

The neonatal dolutegravir regimen evaluated in this study offers several advantages, including potent antiviral activity, a high resistance barrier, simplified dosing, and use of globally available paediatric dispersible tablets. The early-life dolutegravir exposures observed in IMPAACT 2023 are consistent with exposures associated with virological efficacy in older infants and adults, supporting dolutegravir as a candidate component of neonatal presumptive HIV therapy or early treatment strategies.

PETITE-DTG evaluated the same regimen (dolutegravir 5 mg every 48 h during the first 2 weeks of life followed by once-daily dosing until week 4) in South African term neonates born to women receiving long-term dolutegravir-based therapy (median duration 35 months). Infants in the multidose stage were randomly assigned to receive dispersible tablets or oral films, with reassuring pharmacokinetic, safety, and acceptability findings for both formulations.^12^ IMPAACT 2023 provides complementary multicountry, multisite data in infants with and without recent antepartum maternal dolutegravir exposure and used single-dose pharmacokinetic data and modelling to select the multidose regimen.^15,22^ Concordant pharmacokinetic and safety findings across the two independent studies strengthen confidence in the neonatal regimen, and each provides distinct information regarding maternal dolutegravir exposure, formulation, and implementation. Together, findings from IMPAACT 2023 and PETITE-DTG informed WHO recommendations in 2026 supporting dolutegravir use from birth in term infants weighing at least 2 kg.^23^ Clinical use of this regimen remains risk-specific and context-specific and should follow current national and international recommendations. Dolutegravir was first approved for adults in 2013, whereas evidence-based dosing from birth became available more than a decade later. This delay highlights persistent inequities in generating pharmacology and age-appropriate formulation data for pregnant people and neonates and underscores the need to incorporate these populations earlier in antiretroviral development.

The neonatal dosing regimen evaluated in IMPAACT 2023 and PETITE-DTG is not recommended for preterm infants. Preterm neonates have impaired bilirubin–albumin binding, lower UGT1A1 activity, and greater physiological variability in drug clearance, increasing susceptibility to bilirubin-induced neurological dysfunction from drug displacement interactions with drug accumulation.^24–26^ Because clinical assays to measure unbound unconjugated bilirubin are not routinely available, accurately assessing bilirubin toxicity risk is challenging when integrase inhibitors are administered in this population. Safe and effective use of dolutegravir in preterm infants will require careful evaluation of dolutegravir pharmacokinetic and safety data from dedicated studies in preterm infants.

Limitations of this study include a modest sample size typical of phase 1 neonatal trials, imprecision around safety event estimates, and limited ability to capture long-term outcomes. Some infants lacked complete pharmacokinetic profiles because of missed 48-h samples, although sensitivity analyses confirmed the robustness of the pharmacokinetic conclusions. Because intensive pharmacokinetic sampling was not done immediately before the day-14 dosing transition, exposure over the full 48-hourly dosing phase was not directly characterised. However, week 2 cannot be assumed to represent maximal exposure because accumulation occurs alongside rapid maturation of clearance. The study was not designed or powered to assess sex-based differences in pharmacokinetics or safety. Finally, generalisability to preterm infants or term infants with birthweight less than 2 kg remains unknown and requires future study.

In conclusion, IMPAACT 2023 demonstrates that dolutegravir 5 mg dispersible tablets administered every other day until day 14 of life, then once daily until week 4 of life, provides acceptable exposures and is well tolerated in full-term neonates with a birthweight of at least 2 kg born to women living with HIV-1. Together with the independent, concordant PETITE-DTG findings, these results strengthen support for this neonatal dolutegravir dosing regimen and its consideration in presumptive HIV therapy and early treatment strategies.

## Supplementary Material

Supplementary appendix

## Figures and Tables

**Figure 1:**
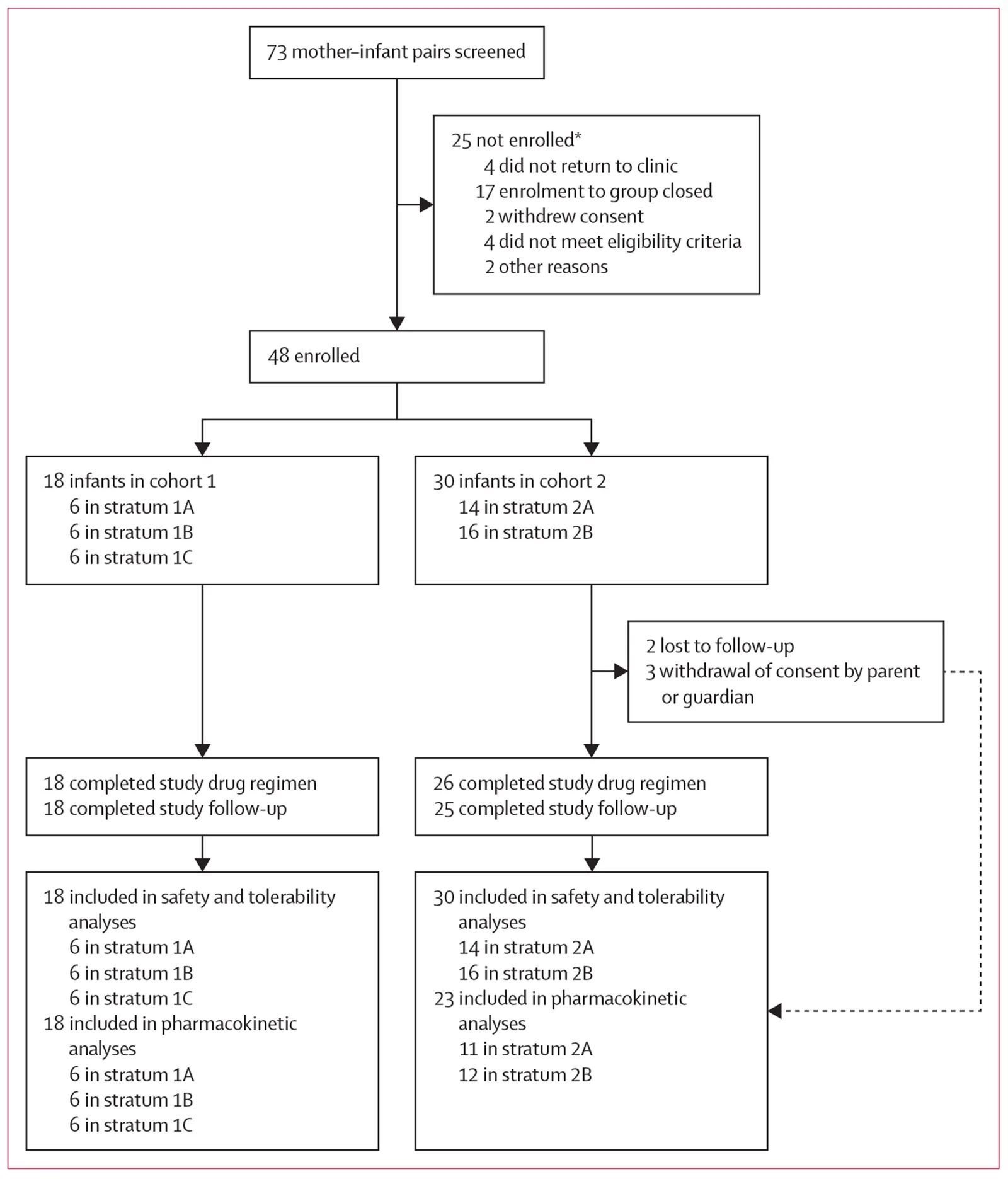
Trial profile All enrolled infants received at least one dose of study drug. *A mother–infant pair could have more than one reason for non-enrolment.

**Figure 2:**
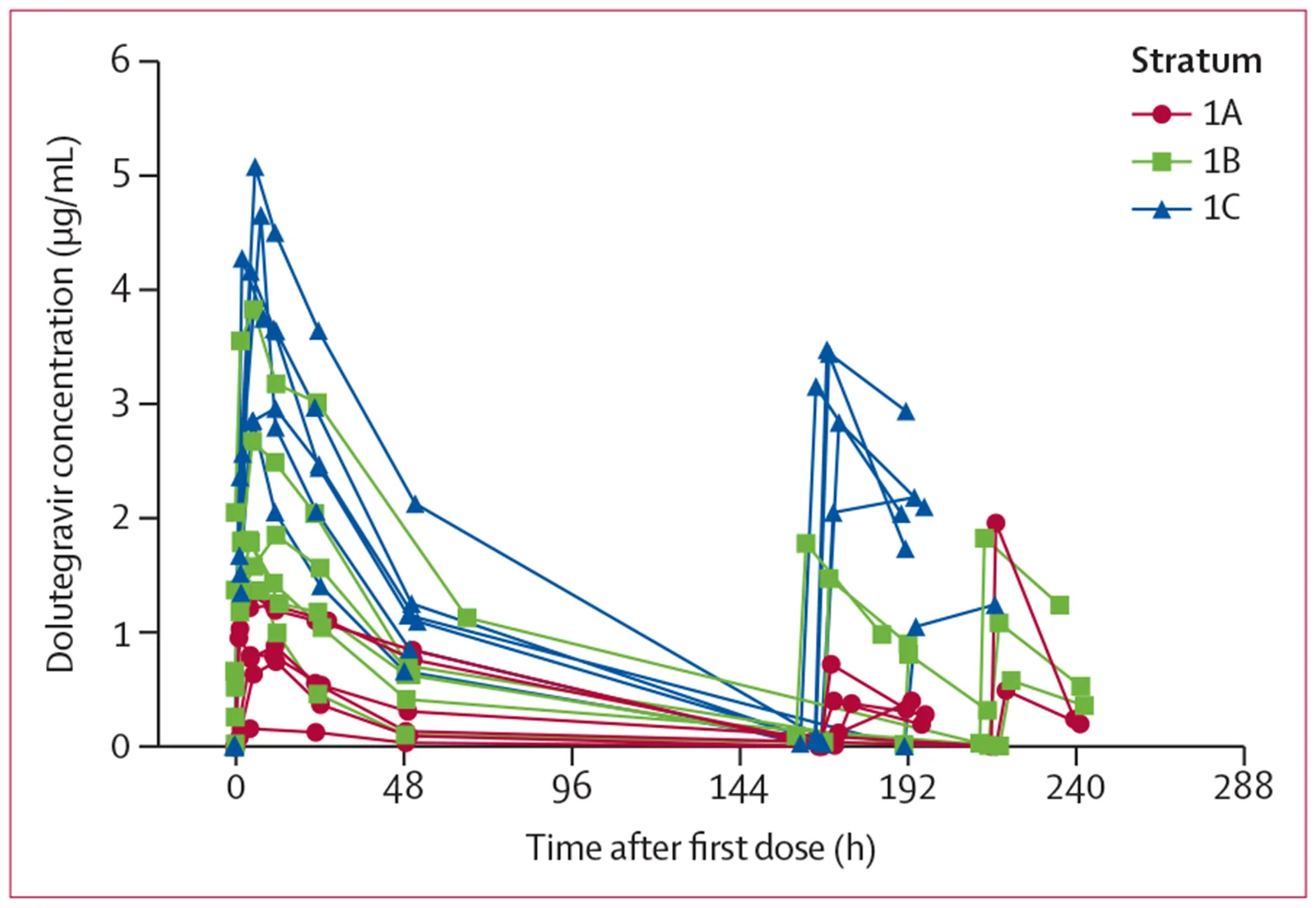
Individual dolutegravir concentration–time profiles in cohort 1 by stratum Each symbol represents an individual participant, with lines connecting serial samples. Cohort 1 was divided into three strata: 1A (dolutegravir-naive, 0·5 mg/kg liquid suspension), 1B (dolutegravir-exposed, 0·5 mg/kg liquid suspension), and 1C (dolutegravir-naive, 5 mg dispersible tablet).

**Figure 3:**
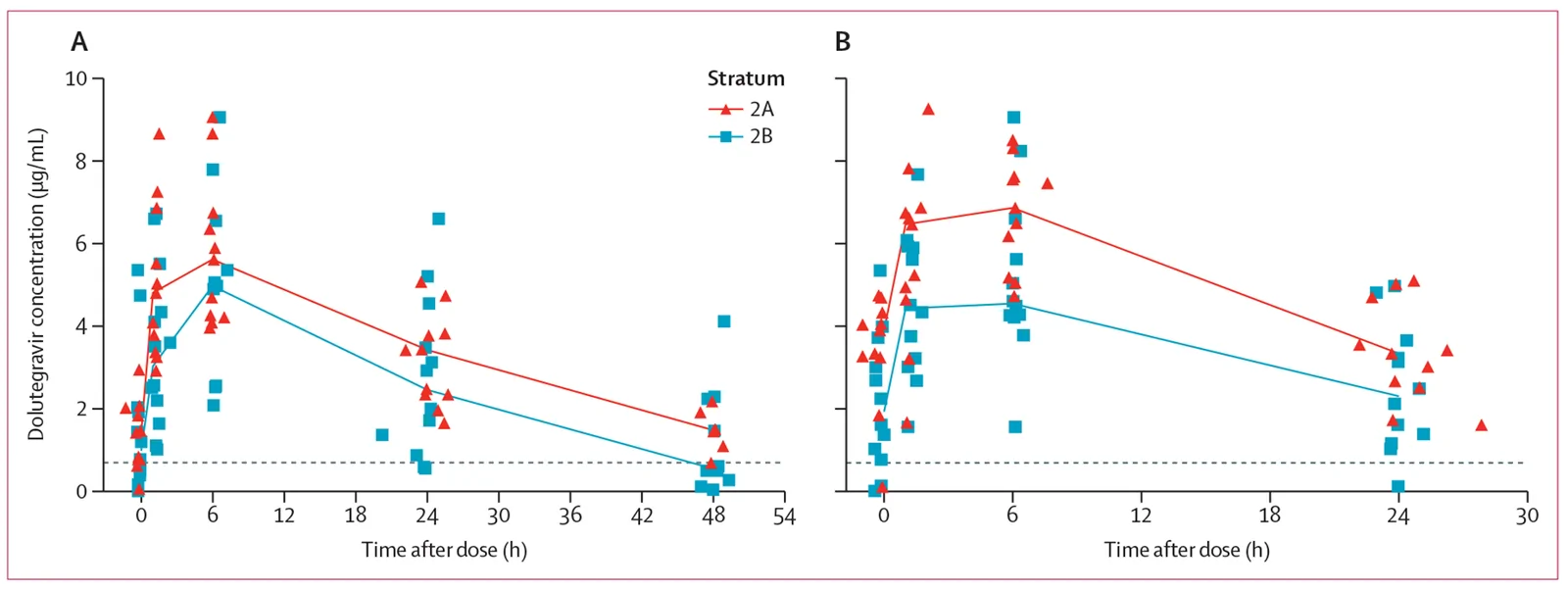
Dolutegravir concentrations in cohort 2 Plasma dolutegravir concentrations in cohort 2 at 7 days after initial dose (A) and at week 4 of life (B) in neonates exposed to HIV-1. Red triangles represent dolutegravir-naive infants (stratum 2A) and blue squares represent infants with in utero dolutegravir exposure (stratum 2B). Solid lines indicate median concentrations. Horizontal dashed line denotes the minimum target concentration (0·697 μg/mL).

**Table 1:** Infant characteristics by stratum

	Cohort 1			Cohort 2	
	Stratum 1A (N=6)	Stratum 1B (N=6)	Stratum 1C (N=6)	Stratum 2A (N=14)	Stratum 2B (N=16)
Age at entry, days	2·3 (1·21)	2·8 (1·47)	3·8 (0·98)	3·1 (1·61)	3·2 (1·47)

Sex					
Female	5	2	3	10	10
Male	1	4	3	4	6

Gestational age, weeks	39·24 (0·98)	38·45 (0·44)	39·07 (1·21)	39·06 (0·60)	39·17 (1·50)

Birthweight, kg	2·88 (0·35)	3·05 (0·20)	3·08 (0·29)	3·08 (0·33)	3·11 (0·35)

Race					
Asian	2	5	3	4	2
Black or African American	4	1	3	5	13
Multiple	0	0	0	1	0
Unknown	0	0	0	1	1
White	0	0	0	3	0

Country					
South Africa	1	0	1	0	13
Thailand	2	5	3	4	2
USA	3	1	2	10	1

Breastfeeding	0	0	2	3	11

Data are mean (SD) or n. Strata 1A, 1C, and 2A were dolutegravir-naive; strata 1B and 2B were dolutegravir-exposed.

**Table 2:** Dolutegravir pharmacokinetic parameters in cohort 2

	Stratum	N	AUC_0–τ_ (μg·h/mL)	C_trough_ (μg/mL)	C_max_ (μg/mL)	t_1/2_ (h)
7 days after initial dose						
Receiving dose every 48 h	2A and 2B (pooled)	21	129 (98·1–169)	0·987 (0·605–1·61)	5·07 (4·21–6·12)	19·7 (16·6–23·4)
Receiving dose every 24 h	2A	2	73·4–110	2·36–3·45	3·97–6·36	20·1–24·0
Week 4 of life	2A and 2B (pooled)	23	97·3 (79·5–119)	2·39 (1·70–3·36)	5·72 (4·83–6·76)	16·8 (13·5–20·8)

Values for infants receiving dose every 24 h (n=2) are presented as observed range; all other measures are geometric mean (95% CI). Values are reported to three significant figures. AUC_0–τ_= area under the concentration–time curve over one dosing interval. C_trough_=concentration of dolutegravir at the end of the dosing interval. C_max_=maximum dolutegravir concentration. t_1/2_=terminal elimination half-life of dolutegravir.

## Data Availability

This study’s data cannot be made publicly available due to ethical restrictions in the study’s informed consent documents and in the IMPAACT Network’s approved human subjects protection plan; public availability may compromise participant confidentiality. However, data, including participant data with partially identifying information, are available to interested researchers upon request to the IMPAACT Statistical and Data Management Center’s Data Access Committee (sdac.data@fstrf.org) with the agreement of the IMPAACT Network.

## Research in context

### Evidence before this study

We searched PubMed and reference lists of relevant articles for studies published from database inception up to April 7, 2026, with no language restrictions. Search terms included “dolutegravir”, “DTG”, “neonate”, “newborn”, “infant”, “HIV”, “pharmacokinetics”, “safety”, “in utero exposure”, “maternal exposure”, and “raltegravir”. We included clinical studies, pharmacokinetic analyses, and modelling studies reporting dolutegravir use, neonatal dolutegravir washout after antepartum maternal dolutegravir exposure, or integrase inhibitor dosing and safety in neonates aged less than 28 days. We excluded studies limited to older infants or children without neonatal data, non-clinical studies, and reports without pharmacokinetic or safety information relevant to dosing from birth. Evidence for neonatal dolutegravir dosing consisted initially mainly of washout pharmacokinetic data after antepartum maternal dolutegravir exposure and population pharmacokinetic modelling and simulation studies; subsequent multidose neonatal data, including from PETITE-DTG, have expanded the evidence base. At the time of the literature search, raltegravir was the only integrase inhibitor approved for use from birth, but it requires a more complex neonatal dosing schedule and has a lower barrier to resistance than dolutegravir. Overall, the available evidence was limited by small sample sizes, non-randomised designs, indirectness for direct dosing from birth, and imprecision; no formal meta-analysis was appropriate.

### Added value of this study

IMPAACT 2023 provides prospective pharmacokinetic, safety, and tolerability data for dolutegravir administered from birth in term neonates weighing at least 2 kg. The study used an adaptive design in which single-dose pharmacokinetic and safety data from cohort 1 informed population pharmacokinetic modelling and simulation to select the chronic regimen evaluated in cohort 2. The results support use of dolutegravir 5 mg dispersible tablets every 48 h during the first 14 days of life, followed by once-daily dosing up to 4 weeks or 6 weeks of age, based on local standard of care, in term neonates with and without antepartum maternal dolutegravir exposure.

### Implications of all the available evidence

These findings strengthen the evidence base for dolutegravir use from birth in term neonates weighing at least 2 kg. The results support consideration of dolutegravir as a component of neonatal presumptive HIV therapy or early treatment strategies and help inform guideline and regulatory discussions on integrase inhibitor use in the immediate neonatal period. Additional studies are needed in preterm neonates, infants weighing less than 2 kg at birth, and the broader clinical implementation setting.
