# Supplementary material for: Pharmacokinetics and safety of dolutegravir in term neonates exposed to HIV-1 (IMPAACT 2023): a phase 1, open-label, multicentre study
Source: Lancet HIV. Author manuscript; Available in PMC 2026 Nov 5. (PMC13738351; doi:10.1016/S2352-3018(26)00226-2)
Supplement: Supplementary file 1 [file NIHMS2218694-supplement-Supplementary_appendix.pdf]

THE LANCET
HIV

Supplementary appendix

This appendix formed part of the original submission and has been peer reviewed. We post it as supplied by the authors.

Supplement to: Momper JD, Wang J, Bradford S, et al. Pharmacokinetics and safety of dolutegravir in term neonates exposed to HIV-1 (IMPAACT 2023): a phase 1, open-label, multicentre study. *Lancet HIV* 2026; published online Oct 21. https://doi.org/10.1016/S2352-3018(26)00226-2.

Table of Contents

Figure S1. Protocol Overview.......... 2

Sensitivity and Supplementary Analyses.......... 3

Table S1. Dolutegravir Pharmacokinetic Parameters in Cohort 2 at 7 Days Post Initial Dose Among Infants Receiving Q48H Dosing.......... 4

Table S2. Summary of Cohort 2 Drug-Related Safety Failure Events Through Two Weeks After Dolutegravir Discontinuation.......... 4

Table S3. Summary of Cohort 2 Drug-Related Safety Failure Events Through Week 16.......... 4

Table S4. Summary of Cohort 2 Safety Failure Events Through Two Weeks After Dolutegravir Discontinuation.......... 5

Table S5. Summary of Cohort 2 Safety Failure Events Through Week 16.......... 5

Table S6. Cohort 2 Estimated Probability of Experiencing At Least One Safety Failure Event Through Week 16.......... 5

Table S7. Adverse Events Through Week 16.......... 6

Table S8. IMPAACT 2023 Study Team.......... 14

Table S9. Approvals for international IMPAACT 2023 sites.......... 16

Figure S1. Protocol Overview. Full protocol is available on the IMPAACT web site (https://www.impaactnetwork.org/sites/default/files/2024-04/IMPAACT2023_Protocol_V2_23MAR22_CM1_12JUL23_CM2_03APR24%20%28002%29_0.pdf)

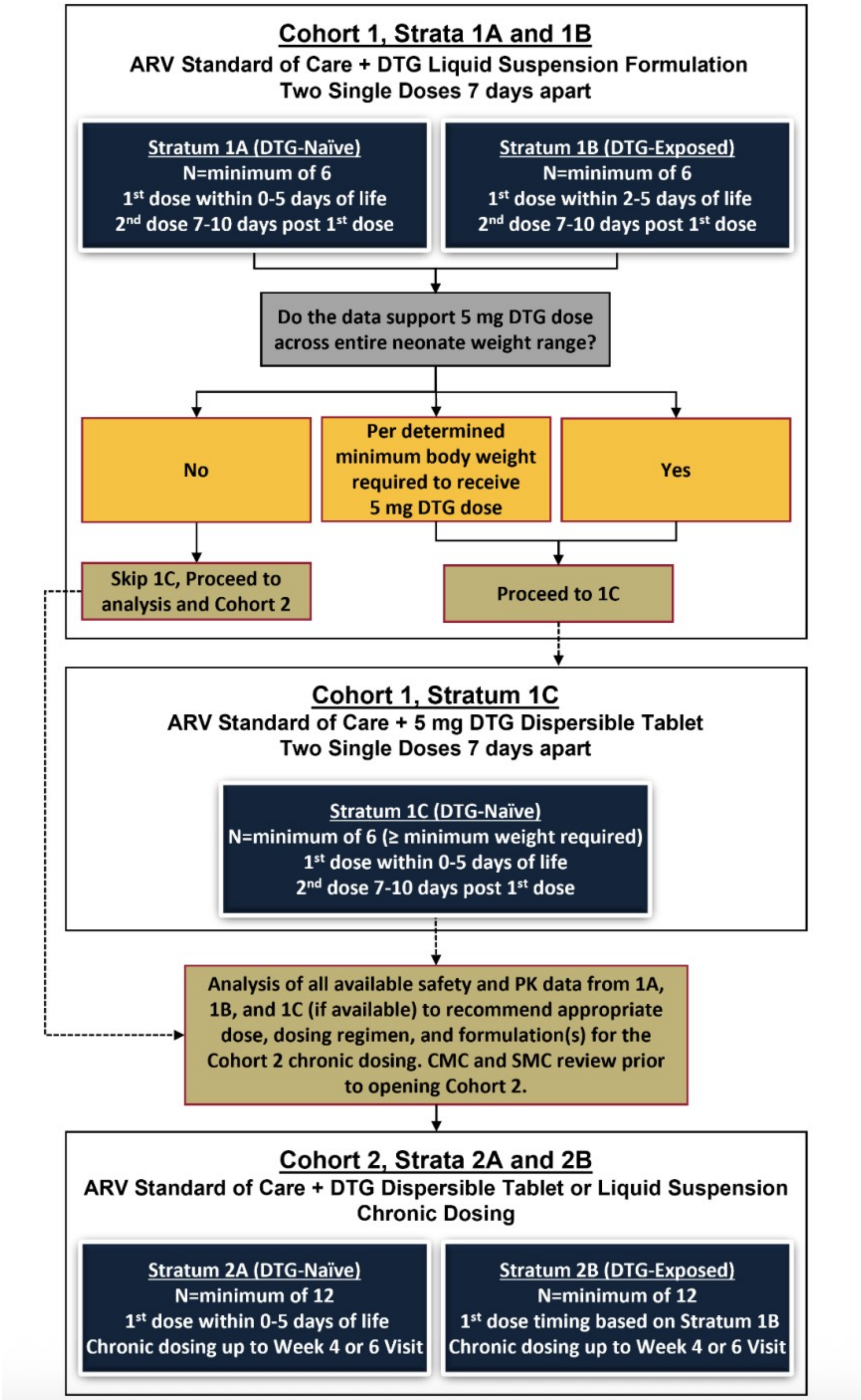

Sensitivity and Supplementary Analyses

Pharmacokinetics

In Cohort 2, DTG 5 mg dispersible tablets were administered every 48 h until day 14 of life, followed by once-daily dosing. Infants received their first DTG dose at 0–5 days of life. PK sampling was performed 7 days after DTG initiation and at week 4 of life. For infants still receiving every-48-h dosing at the day 7 visit, samples were collected predose and at 1–2 h, 6–10 h, 22–26 h, and 48 h postdose, with the 48-h sample obtained before administration of the next dose. For infants who had transitioned to once-daily dosing, samples were collected predose and at 1–2 h, 6–10 h, and 22–26 h postdose; i.e., these infants did not have a 48-hour trough sample collected. These infants were included in the main pharmacokinetic analysis (Table 2 and Table S1, first row) by interpolating C48 from preceding terminal-phase concentrations. In a sensitivity analysis (Table S1, second row), these infants were excluded.

Safety

Per the Primary Statistical Analysis Plan, the main safety analysis for both the primary and secondary safety outcome measures was to use single imputation under the best-case scenario (assume no safety event after premature study discontinuation). Since it was recognized that this approach may underestimate adverse event rates, sensitivity analyses were also to be performed using complete-case analysis (exclude participants with any missing safety data) and single imputation under the worst-case scenario (impute safety event after premature study discontinuation).

In Cohort 1, none of the 18 infants experienced drug-related safety failure or safety failure and none discontinued study follow-up prematurely. The 90% confidence intervals (CIs) for the probability of experiencing a drug-related safety failure or safety failure (regardless of attribution to study drug) through two weeks after dolutegravir discontinuation and through Week 16 were all the same, 0-15%, in the main, complete-case, and worst-case sensitivity analyses.

In Cohort 2, none of the 30 infants experienced drug-related safety failure. Four infants experienced safety failure within two weeks after dolutegravir discontinuation and a total of seven infants experienced safety failure by Week 16. Three infants discontinued study follow-up prematurely within two weeks after dolutegravir discontinuation and a total of five infants discontinued study follow-up prematurely before Week 16. Tables S2 and S3 show the estimated probabilities and corresponding 90% CIs for drug-related safety failure through two weeks after dolutegravir discontinuation and through Week 16, respectively, from the main, complete-case, and worst-case analyses. Tables S4 and S5 show the estimated probabilities and corresponding 90% CIs for safety failure (regardless of attribution to study drug) through two weeks after dolutegravir discontinuation and through Week 16, respectively, from the main, complete-case, and worst-case analyses.

In the supplementary time-to-event analyses for Cohort 2, as there were no drug-related safety failures, the CIs could not be estimated with the planned analysis method and are not shown. Kaplan-Meier estimates and CIs for the probability of a safety failure through Week 16 are shown in Table S6.

Tolerability

For Cohort 2, the primary tolerability outcome was the proportion of infants who experienced protocol-defined problems taking the study drug (refused, vomited or spit up, gagged or had other difficulties) for "approximately half of the doses" or "most doses". No infants experienced this outcome, so the 90% CI for the probability of not being able to tolerate dolutegravir was 0 to 9.5%. In a supplementary analysis, the tolerability outcome definition was expanded to include experiencing problems taking the study drug "infrequently" in addition to "approximately half the doses" and "most doses". Since three of the 30 infants experienced infrequent vomiting/spitting up or gagging, the estimated probability of not being able to tolerate dolutegravir increased to 10% (90% CI 2.8 to 23.9%) in this supplemental analysis.

Table S1. Dolutegravir Pharmacokinetic Parameters in Cohort 2 at 7 Days Post Initial Dose Among Infants Receiving Q48H Dosing

	$AUC_{0-\tau}$ (µg·h/mL)	C_{trough} (µg/mL)	C_{max} (µg/mL)	$T_{½}$ (h)
Main Analysis (n=21)	129 (98·1–169)	0·987 (0·605–1·61)	5·07 (4·21–6·12)	19·7 (16·6–23·4)
Sensitivity Analysis* (n=16)	119 (84·3–167)	0·838 (0·449–1·57)	4·85 (3·83–6·14)	18·4 (14·8–22·9)

*Excludes the 5 participants (3 in Stratum 2A and 2 in Stratum 2B) with PK sampling performed at the last dose of Q48H dosing (first dose of Q24H dosing), so a 48-hour sample was not collected

S2. Summary of Cohort 2 Drug-Related Safety Failure Events Through Two Weeks After Dolutegravir Discontinuation

	Stratum 2A (N=14) (N_C=13)		Stratum 2B (N=16) (N_C=14)		Total (N=30) (N_C=27)	
	n (%)	90% CI (%)	n (%)	90% CI (%)	n (%)	90% CI (%)
Main analysis	0 (0)	(0, 19·3)	0 (0)	(0, 17·1)	0 (0)	(0, 9·5)
Complete-case analysis	0 (0)	(0, 20·6)	0 (0)	(0, 19·3)	0 (0)	(0, 10·5)
Worst-case analysis	1 (7·1)	(0·37, 29·67)	2 (12·5)	(2·27, 34·38)	3 (10·0)	(2·78, 23·86)

N = Number of participants in each cohort/stratum
N_C = Number of participants in each cohort/stratum for complete case sensitivity analysis
n (%) = Number (percent) of participants in each cohort/stratum who experienced at least one such event (with respect to the number of participants in each cohort/stratum for complete case sensitivity analysis)
90% CI = Exact 90% Confidence Interval (Clopper-Pearson)
Grade: 3 = Severe, 4 = Potentially Life-Threatening, 5 = Death

S3. Summary of Cohort 2 Drug-Related Safety Failure Events Through Week 16

	Stratum 2A (N=14) (N_C=12)		Stratum 2B (N=16) (N_C=13)		Total (N=30) (N_C=25)	
	n (%)	90% CI (%)	n (%)	90% CI (%)	n (%)	90% CI (%)
Main analysis	0 (0)	(0, 19·3)	0 (0)	(0, 17·1)	0 (0)	(0, 9·5)
Complete-case analysis	0 (0)	(0, 22·1)	0 (0)	(0, 20·6)	0 (0)	(0, 11·3)
Worst-case analysis	2 (14·3)	(2.6, 38·5)	3 (18·8)	(5·3, 41·7)	5 (16·7)	(6·8, 31·9)

N = Number of participants in each cohort/stratum
N_C = Number of participants in each cohort/stratum for complete case sensitivity analysis
n (%) = Number (percent) of participants in each cohort/stratum who experienced at least one such event (with respect to
the number of participants in each cohort/stratum for complete case sensitivity analysis)
90% CI = Exact 90% Confidence Interval (Clopper-Pearson)
Grade: 3 = Severe, 4 = Potentially Life-Threatening, 5 = Death

Table S4. Summary of Cohort 2 Safety Failure Events Through Two Weeks After Dolutegravir Discontinuation

	Stratum 2A (N=14) (N_C=13)		Stratum 2B (N=16) (N_C=14)		Total (N=30) (N_C=27)	
	**n (%)**	**90% CI (%)**	**n (%)**	**90% CI (%)**	**n (%)**	**90% CI (%)**
Main analysis	3 (21·4)	(6·1, 46·6)	1 (6·3)	(0·32, 26·4)	4 (13·3)	(4·7, 28·0)
Complete-case analysis	3 (23·1)	(6·6, 49·5)	1 (7·1)	(0·37, 29·7)	4 (14·8)	(5·2, 30·8)
Worst-case analysis	4 (28·6)	(10·4, 54·0)	3 (18·8)	(5·31, 41·7)	7 (23·3)	(11·5, 39·4)

N = Number of participants in each cohort/stratum
N_C = Number of participants in each cohort/stratum for complete case sensitivity analysis
n (%) = Number (percent) of participants in each cohort/stratum who experienced at least one such event (with respect to
the number of participants in each cohort/stratum for complete case sensitivity analysis)
90% CI = Exact 90% Confidence Interval (Clopper-Pearson)
Grade: 3 = Severe, 4 = Potentially Life-Threatening, 5 = Death

Table S5. Summary of Cohort 2 Safety Failure Events Through Week 16

	Stratum 2A (N=14) (N_C=12)		Stratum 2B (N=16) (N_C=13)		Total (N=30) (N_C=25)	
	**n (%)**	**90% CI (%)**	**n (%)**	**90% CI (%)**	**n (%)**	**90% CI (%)**
Main analysis	4 (28·6)	(10·4, 54·0)	3 (18·8)	(5·3, 41·7)	7 (23·3)	(11·5, 39·5)
Complete-case analysis	4 (33·3)	(12·3, 60·9)	3 (23·1)	(6·6, 49·5)	7 (28·0)	(13·9, 46·2)
Worst case-analysis	6 (42·9)	(20·6, 67·5)	6 (37·5)	(17·8, 60·9)	12 (40·0)	(24·9, 56·6)

N = Number of participants in each cohort/stratum
N_C = Number of participants in each cohort/stratum for complete case sensitivity analysis
n (%) = Number (percent) of participants in each cohort/stratum who experienced at least one such event (with respect to
the number of participants in each cohort/stratum for complete case sensitivity analysis)
90% CI = Exact 90% Confidence Interval (Clopper-Pearson)
Grade: 3 = Severe, 4 = Potentially Life-Threatening, 5 = Death

Table S6. Cohort 2 Estimated Probability of Experiencing At Least One Safety Failure Event Through Week 16

	Cohort 2 (N=30)					
**Analysis Visit**	**n**	**Cumulative Number of Participants with Events**	**Cumulative Number of Participants Censored**	**KM Estimate**	**90% CI**
Week 4	25	2	3	0·07	(0·02, 0·20)
Week 6	23	4	3	0·14	(0·06, 0·29)
Week 8	22	5	3	0·18	(0·09, 0·34)
Week 16	20	6	4	0·22	(0·12, 0·38)

N = Number of participants in cohort
n = Number at risk at the beginning of the visit window
KM Estimate = Kaplan-Meier estimate of the probability of experiencing at least one safety failure event at the time point
90% CI = log-log Confidence Interval
Time points listed use the lower endpoint of the safety visit window.

Table S7. Adverse Events Through Week 16

Frequency Threshold Above Which Other Adverse Events are Reported: 0%

	Cohort 1 Stratum 1A	Cohort 1 Stratum 1B	Cohort 1 Stratum 1C	Cohort 1 Total	Cohort 2 Stratum 2A	Cohort 2 Stratum 2B
	Affected/At Risk (%)	Affected/At Risk (%)	Affected/At Risk (%)	Affected/At Risk (%)	Affected/ At Risk (%)	Affected/At Risk (%)
Total	4/6 (66.67%)	5/6 (83.33%)	5/6 (83.33%)	14/18 (77.78%)	12/14 (85.71%)	14/16 (87.5%)
Blood and lymphatic system disorders						
Anaemia A †	0/6 (0%)	0/6 (0%)	0/6 (0%)	0/18 (0%)	2/14 (14.29%)	0/16 (0%)
Neutropenia A †	1/6 (16.67%)	1/6 (16.67%)	0/6 (0%)	2/18 (11.11%)	1/14 (7.14%)	0/16 (0%)
Congenital, familial and genetic disorders						
Congenital umbilical hernia A †	0/6 (0%)	0/6 (0%)	0/6 (0%)	0/18 (0%)	0/14 (0%)	1/16 (6.25%)
Gastrointestinal disorders						
Abdominal hernia A †	0/6 (0%)	0/6 (0%)	0/6 (0%)	0/18 (0%)	1/14 (7.14%)	0/16 (0%)
Anal fissure A †	0/6 (0%)	0/6 (0%)	0/6 (0%)	0/18 (0%)	1/14 (7.14%)	0/16 (0%)
Constipation A †	1/6 (16.67%)	0/6 (0%)	0/6 (0%)	1/18 (5.56%)	0/14 (0%)	0/16 (0%)
Flatulence A †	0/6 (0%)	0/6 (0%)	0/6 (0%)	0/18 (0%)	0/14 (0%)	1/16 (6.25%)
Infantile diarrhoea A †	0/6 (0%)	0/6 (0%)	0/6 (0%)	0/18 (0%)	0/14 (0%)	2/16 (12.5%)
Infantile vomiting A †	0/6 (0%)	0/6 (0%)	0/6 (0%)	0/18 (0%)	2/14 (14.29%)	0/16 (0%)
General disorders						
Fever neonatal A †	0/6 (0%)	0/6 (0%)	0/6 (0%)	0/18 (0%)	1/14 (7.14%)	0/16 (0%)
Swelling A †	0/6 (0%)	1/6 (16.67%)	0/6 (0%)	1/18 (5.56%)	0/14 (0%)	0/16 (0%)
Hepatobiliary disorders						
Hyperbilirubinaemia A †	0/6 (0%)	1/6 (16.67%)	0/6 (0%)	1/18 (5.56%)	0/14 (0%)	0/16 (0%)

	Cohort 1 Stratum 1A	Cohort 1 Stratum 1B	Cohort 1 Stratum 1C	Cohort 1 Total	Cohort 2 Stratum 2A	Cohort 2 Stratum 2B
	Affected/At Risk (%)	Affected/At Risk (%)	Affected/At Risk (%)	Affected/At Risk (%)	Affected/ At Risk (%)	Affected/At Risk (%)
Hyperbilirubinaemia neonatal [A] †	1/6 (16.67%)	1/6 (16.67%)	0/6 (0%)	2/18 (11.11%)	1/14 (7.14%)	1/16 (6.25%)
Hypertransaminasaemia [A] †	0/6 (0%)	0/6 (0%)	0/6 (0%)	0/18 (0%)	1/14 (7.14%)	0/16 (0%)
Ocular icterus [A] †	0/6 (0%)	0/6 (0%)	0/6 (0%)	0/18 (0%)	1/14 (7.14%)	0/16 (0%)
Infections and infestations						
Body tinea [A] †	0/6 (0%)	0/6 (0%)	0/6 (0%)	0/18 (0%)	0/14 (0%)	1/16 (6.25%)
Bronchiolitis [A] †	0/6 (0%)	0/6 (0%)	0/6 (0%)	0/18 (0%)	1/14 (7.14%)	0/16 (0%)
Bronchitis [A] †	0/6 (0%)	0/6 (0%)	0/6 (0%)	0/18 (0%)	0/14 (0%)	1/16 (6.25%)
Candida nappy rash [A] †	0/6 (0%)	0/6 (0%)	0/6 (0%)	0/18 (0%)	1/14 (7.14%)	0/16 (0%)
Fungal skin infection [A] †	1/6 (16.67%)	0/6 (0%)	0/6 (0%)	1/18 (5.56%)	0/14 (0%)	0/16 (0%)
Gastroenteritis viral [A] †	0/6 (0%)	0/6 (0%)	0/6 (0%)	0/18 (0%)	0/14 (0%)	1/16 (6.25%)
Nasopharyngitis [A] †	0/6 (0%)	0/6 (0%)	0/6 (0%)	0/18 (0%)	1/14 (7.14%)	1/16 (6.25%)
Ophthalmia neonatorum [A] †	0/6 (0%)	0/6 (0%)	0/6 (0%)	0/18 (0%)	1/14 (7.14%)	0/16 (0%)
Oral candidiasis [A] †	0/6 (0%)	0/6 (0%)	1/6 (16.67%)	1/18 (5.56%)	2/14 (14.29%)	0/16 (0%)
Otitis media [A] †	0/6 (0%)	0/6 (0%)	0/6 (0%)	0/18 (0%)	0/14 (0%)	1/16 (6.25%)
Respiratory tract infection [A] †	0/6 (0%)	0/6 (0%)	0/6 (0%)	0/18 (0%)	0/14 (0%)	1/16 (6.25%)
Tinea faciei [A] †	1/6 (16.67%)	0/6 (0%)	0/6 (0%)	1/18 (5.56%)	0/14 (0%)	0/16 (0%)
Upper respiratory tract infection [A] †	0/6 (0%)	0/6 (0%)	1/6 (16.67%)	1/18 (5.56%)	1/14 (7.14%)	0/16 (0%)
Viral upper respiratory tract infection [A] †	0/6 (0%)	0/6 (0%)	0/6 (0%)	0/18 (0%)	0/14 (0%)	1/16 (6.25%)
Investigations						
Alanine aminotransferase increased [A] †	1/6 (16.67%)	1/6 (16.67%)	0/6 (0%)	2/18 (11.11%)	1/14 (7.14%)	0/16 (0%)
Aspartate aminotransferase increased [A] †	1/6 (16.67%)	0/6 (0%)	0/6 (0%)	1/18 (5.56%)	2/14 (14.29%)	0/16 (0%)

	Cohort 1 Stratum 1A	Cohort 1 Stratum 1B	Cohort 1 Stratum 1C	Cohort 1 Total	Cohort 2 Stratum 2A	Cohort 2 Stratum 2B
	Affected/At Risk (%)	Affected/At Risk (%)	Affected/At Risk (%)	Affected/At Risk (%)	Affected/ At Risk (%)	Affected/At Risk (%)
Blood albumin decreased A †	0/6 (0%)	0/6 (0%)	0/6 (0%)	0/18 (0%)	1/14 (7.14%)	0/16 (0%)
Blood bilirubin increased A †	0/6 (0%)	1/6 (16.67%)	0/6 (0%)	1/18 (5.56%)	5/14 (35.71%)	4/16 (25%)
Blood creatinine increased A †	0/6 (0%)	0/6 (0%)	0/6 (0%)	0/18 (0%)	2/14 (14.29%)	3/16 (18.75%)
Blood potassium increased A †	0/6 (0%)	0/6 (0%)	0/6 (0%)	0/18 (0%)	2/14 (14.29%)	0/16 (0%)
Cardiac murmur A †	0/6 (0%)	0/6 (0%)	0/6 (0%)	0/18 (0%)	1/14 (7.14%)	0/16 (0%)
Haemoglobin decreased A †	2/6 (33.33%)	3/6 (50%)	5/6 (83.33%)	10/18 (55.56%)	6/14 (42.86%)	9/16 (56.25%)
Neutrophil count decreased A †	1/6 (16.67%)	1/6 (16.67%)	3/6 (50%)	5/18 (27.78%)	3/14 (21.43%)	5/16 (31.25%)
Weight decreased A †	0/6 (0%)	0/6 (0%)	0/6 (0%)	0/18 (0%)	1/14 (7.14%)	0/16 (0%)
White blood cell count decreased A †	0/6 (0%)	0/6 (0%)	0/6 (0%)	0/18 (0%)	0/14 (0%)	3/16 (18.75%)
Metabolism and nutrition disorders						
Dehydration A †	0/6 (0%)	0/6 (0%)	0/6 (0%)	0/18 (0%)	0/14 (0%)	1/16 (6.25%)
Pregnancy, puerperium and perinatal conditions						
Jaundice neonatal A †	0/6 (0%)	0/6 (0%)	0/6 (0%)	0/18 (0%)	1/14 (7.14%)	0/16 (0%)
Leukopenia neonatal A †	0/6 (0%)	0/6 (0%)	0/6 (0%)	0/18 (0%)	1/14 (7.14%)	0/16 (0%)
Umbilical granuloma A †	0/6 (0%)	0/6 (0%)	0/6 (0%)	0/18 (0%)	0/14 (0%)	1/16 (6.25%)
Respiratory, thoracic and mediastinal disorders						
Cough A †	0/6 (0%)	0/6 (0%)	0/6 (0%)	0/18 (0%)	1/14 (7.14%)	1/16 (6.25%)
Hypoxia A †	0/6 (0%)	0/6 (0%)	0/6 (0%)	0/18 (0%)	1/14 (7.14%)	0/16 (0%)
Nasal congestion A †	0/6 (0%)	0/6 (0%)	1/6 (16.67%)	1/18 (5.56%)	2/14 (14.29%)	2/16 (12.5%)
Rhinorrhoea A †	0/6 (0%)	0/6 (0%)	1/6 (16.67%)	1/18 (5.56%)	1/14 (7.14%)	0/16 (0%)
Sneezing A †	0/6 (0%)	0/6 (0%)	0/6 (0%)	0/18 (0%)	1/14 (7.14%)	0/16 (0%)

	Cohort 1 Stratum 1A	Cohort 1 Stratum 1B	Cohort 1 Stratum 1C	Cohort 1 Total	Cohort 2 Stratum 2A	Cohort 2 Stratum 2B
	Affected/At Risk (%)	Affected/At Risk (%)	Affected/At Risk (%)	Affected/At Risk (%)	Affected/ At Risk (%)	Affected/At Risk (%)
Skin and subcutaneous tissue disorders						
Acne infantile A †	0/6 (0%)	0/6 (0%)	1/6 (16.67%)	1/18 (5.56%)	0/14 (0%)	0/16 (0%)
Dermatitis diaper A †	0/6 (0%)	0/6 (0%)	0/6 (0%)	0/18 (0%)	3/14 (21.43%)	2/16 (12.5%)
Dry skin A †	0/6 (0%)	0/6 (0%)	1/6 (16.67%)	1/18 (5.56%)	1/14 (7.14%)	0/16 (0%)
Eczema infantile A †	0/6 (0%)	0/6 (0%)	0/6 (0%)	0/18 (0%)	3/14 (21.43%)	2/16 (12.5%)
Erythema A †	0/6 (0%)	0/6 (0%)	0/6 (0%)	0/18 (0%)	1/14 (7.14%)	0/16 (0%)
Macule A †	0/6 (0%)	0/6 (0%)	0/6 (0%)	0/18 (0%)	1/14 (7.14%)	0/16 (0%)
Miliaria A †	0/6 (0%)	1/6 (16.67%)	0/6 (0%)	1/18 (5.56%)	0/14 (0%)	1/16 (6.25%)
Rash A †	0/6 (0%)	0/6 (0%)	0/6 (0%)	0/18 (0%)	1/14 (7.14%)	2/16 (12.5%)
Rash neonatal A †	0/6 (0%)	0/6 (0%)	0/6 (0%)	0/18 (0%)	0/14 (0%)	2/16 (12.5%)
Seborrhoea A †	0/6 (0%)	0/6 (0%)	0/6 (0%)	0/18 (0%)	1/14 (7.14%)	0/16 (0%)
Seborrhoeic dermatitis A †	0/6 (0%)	0/6 (0%)	1/6 (16.67%)	1/18 (5.56%)	1/14 (7.14%)	0/16 (0%)
Skin disorder A †	0/6 (0%)	0/6 (0%)	0/6 (0%)	0/18 (0%)	0/14 (0%)	1/16 (6.25%)
Skin exfoliation A †	0/6 (0%)	0/6 (0%)	0/6 (0%)	0/18 (0%)	1/14 (7.14%)	0/16 (0%)

†Indicates events were collected by systematic assessment.
A Term from vocabulary, MedDRA 28.0

	Cohort 2 Total
	Affected/At Risk (%)
Total	26/30 (86.67%)
Blood and lymphatic system disorders	

	Cohort 2 Total
	Affected/At Risk (%)
Anaemia ^A †	2/30 (6.67%)
Neutropenia ^A †	1/30 (3.33%)
Congenital, familial and genetic disorders	
Congenital umbilical hernia ^A †	1/30 (3.33%)
Gastrointestinal disorders	
Abdominal hernia ^A †	1/30 (3.33%)
Anal fissure ^A †	1/30 (3.33%)
Constipation ^A †	0/30 (0%)
Flatulence ^A †	1/30 (3.33%)
Infantile diarrhoea ^A †	2/30 (6.67%)
Infantile vomiting ^A †	2/30 (6.67%)
General disorders	
Fever neonatal ^A †	1/30 (3.33%)
Swelling ^A †	0/30 (0%)
Hepatobiliary disorders	
Hyperbilirubinaemia ^A †	0/30 (0%)
Hyperbilirubinaemia neonatal ^A †	2/30 (6.67%)
Hypertransaminasaemia ^A †	1/30 (3.33%)
Ocular icterus ^A †	1/30 (3.33%)
Infections and infestations	
Body tinea ^A †	1/30 (3.33%)
Bronchiolitis ^A †	1/30 (3.33%)
Bronchitis ^A †	1/30 (3.33%)

	Cohort 2 Total
	Affected/At Risk (%)
Candida nappy rash ᴬ †	1/30 (3.33%)
Fungal skin infection ᴬ †	0/30 (0%)
Gastroenteritis viral ᴬ †	1/30 (3.33%)
Nasopharyngitis ᴬ †	2/30 (6.67%)
Ophthalmia neonatorum ᴬ †	1/30 (3.33%)
Oral candidiasis ᴬ †	2/30 (6.67%)
Otitis media ᴬ †	1/30 (3.33%)
Respiratory tract infection ᴬ †	1/30 (3.33%)
Tinea faciei ᴬ †	0/30 (0%)
Upper respiratory tract infection ᴬ †	1/30 (3.33%)
Viral upper respiratory tract infection ᴬ †	1/30 (3.33%)
Investigations	
Alanine aminotransferase increased ᴬ †	1/30 (3.33%)
Aspartate aminotransferase increased ᴬ †	2/30 (6.67%)
Blood albumin decreased ᴬ †	1/30 (3.33%)
Blood bilirubin increased ᴬ †	9/30 (30%)
Blood creatinine increased ᴬ †	5/30 (16.67%)
Blood potassium increased ᴬ †	2/30 (6.67%)
Cardiac murmur ᴬ †	1/30 (3.33%)
Haemoglobin decreased ᴬ †	15/30 (50%)
Neutrophil count decreased ᴬ †	8/30 (26.67%)

	Cohort 2 Total
	Affected/At Risk (%)
Weight decreased ^A †	1/30 (3.33%)
White blood cell count decreased ^A †	3/30 (10%)
Metabolism and nutrition disorders	
Dehydration ^A †	1/30 (3.33%)
Pregnancy, puerperium and perinatal conditions	
Jaundice neonatal ^A †	1/30 (3.33%)
Leukopenia neonatal ^A †	1/30 (3.33%)
Umbilical granuloma ^A †	1/30 (3.33%)
Respiratory, thoracic and mediastinal disorders	
Cough ^A †	2/30 (6.67%)
Hypoxia ^A †	1/30 (3.33%)
Nasal congestion ^A †	4/30 (13.33%)
Rhinorrhoea ^A †	1/30 (3.33%)
Sneezing ^A †	1/30 (3.33%)
Skin and subcutaneous tissue disorders	
Acne infantile ^A †	0/30 (0%)
Dermatitis diaper ^A †	5/30 (16.67%)
Dry skin ^A †	1/30 (3.33%)
Eczema infantile ^A †	5/30 (16.67%)
Erythema ^A †	1/30 (3.33%)
Macule ^A †	1/30 (3.33%)
Miliaria ^A †	1/30 (3.33%)
Rash ^A †	3/30 (10%)

	Cohort 2 Total
	Affected/At Risk (%)
Rash neonatal A †	2/30 (6.67%)
Seborrhoea A †	1/30 (3.33%)
Seborrhoeic dermatitis A †	1/30 (3.33%)
Skin disorder A †	1/30 (3.33%)
Skin exfoliation A †	1/30 (3.33%)

†Indicates events were collected by systematic assessment.
A Term from vocabulary, MedDRA 28.0

Table S8. IMPAACT 2023 Study Team

Role	Name	Institutional affiliation
Study Chair	Diana Clarke	Section of Pediatric Infectious Diseases, Boston Medical Center, Boston, Massachusetts, USA
Study Vice-Chair	Mark Mirochnick	Department of Pediatrics, Boston University School of Medicine, Boston, Massachusetts, USA
Clinical Research Manager	Sarah Bradford	FHI 360, Durham, North Carolina, USA
Clinical Research Manager	Iris Mustich	FHI 360, Durham, North Carolina, USA
DAIDS Medical Officer	Ellen Townley	International Maternal Adolescent Pediatric Branch, DAIDS/NIAID/NIH, Rockville, Maryland, USA
DAIDS Medical Officer	Dwight Yin	Division of AIDS, DAIDS/NIAID/NIH, Rockville, Maryland, USA
NICHD Medical Officer	Tara DeYampert	National Institute of Child Health and Human Development (NICHD), NIH
NICHD Medical Officer	Sonia Lee	Maternal and Pediatric Infectious Disease Branch, NICHD/NIH, Bethesda, Maryland, USA
Pharmacologist	Edward Acosta	University of Alabama, Birmingham, Alabama, USA
Pharmacologist	Brookie Best	University of California, San Diego, La Jolla, California, USA
Pharmacologist	Jeremiah Momper	University of California, San Diego, La Jolla, California, USA
Statistician	Evan Goldberg	SDAC, Harvard T.H. Chan School of Public Health, Boston, Massachusetts, USA
Statistician	David Shapiro	Harvard T.H. Chan School of Public Health, Center for Biostatistics in AIDS Research, Boston, Massachusetts, USA
Statistician	Jiajia Wang	Harvard T.H. Chan School of Public Health, Center for Biostatistics in AIDS Research, Boston, Massachusetts, USA
Data Manager	Benjamin Johnston	Frontier Science Foundation, Amherst, New York, USA
Data Manager	Madison O'Brien	Frontier Science Foundation, Amherst, New York, USA
Laboratory Technologist	Dean Soko	Queen Elizabeth Central Hospital, Blantyre, Malawi
Community Program Manager	Martine Harrington-Powell	FHI 360, Durham, North Carolina, USA
Community Program Manager	Jonathan Lucas	FHI 360
Investigator	Maria Leticia Cruz	Hospital dos Servidores do Estado, Rio de Janeiro, Brazil
Investigator	Kathleen Powis	Massachusetts General Hospital, Boston, Massachusetts, USA
Westat Representative	Marsha Johnson	Westat, Rockville, Maryland, USA
DAIDS Protocol Pharmacist	Justine Beck	PAB, DAIDS, NIAID, NIH, Rockville, Maryland, USA
DAIDS Protocol Pharmacist	Kelly Colsh	NIH, NIAID, DAIDS, PAB, Rockville, Maryland, USA
DAIDS Protocol Pharmacist	Andrea McMunn	NIH, NIAID, DAIDS, PAB, Rockville, Maryland, USA
ICAB Representative	Maria Randall	Department of Infectious Diseases, St. Jude Children's Research Hospital, Memphis, Tennessee, USA
Laboratory Data Manager (LDM)	Kyle Whitson	Frontier Science Foundation, Amherst, New York, USA
Laboratory Data Manager (LDM)	Sarah Wojcinski	Frontier Science Foundation, Amherst, New York, USA
Laboratory Specialist	Helty Adisetiyo	Milab Central / IMPAACT Laboratory Center, University of California, Los Angeles, Los Angeles, California, USA
Laboratory Specialist	Ceora Beijer-Spell	IMPAACT Laboratory Center, University of California, Los Angeles, Los Angeles, California, USA

Role	Name	Institutional affiliation
Pharmaceutical Company Representative	Marika Ciuffa	GSK
Pharmaceutical Company Representative	Gregory Gardiner	GSK, United Kingdom
Pharmaceutical Company Representative	Rulan Griesel	ViiV Healthcare, London, United Kingdom
Pharmaceutical Company Representative	Alicia Koblansky	ViiV Healthcare
Pharmaceutical Company Representative	Michael McKenna	GSK, Brentford, United Kingdom
Pharmaceutical Company Representative	Rashmi Mehta	GSK

Table S9. Approvals for international IMPAACT 2023 sites

Country	Study Site	Approval Body	Reference number
South Africa	Umlazi CRS	Biomedical Research Ethics Committee, University of KwaZulu-Natal	BREC/00003792/2022
	Wits RHI Shandukani Research Centre CRS	Human Research Ethics Committee (Medical), University of the Witwatersrand	220508B
	Soweto IMPAACT CRS	Human Research Ethics Committee (Medical), University of the Witwatersrand	220508B
	FAMCRU CRS	Health Research Ethics Committee 2, Stellenbosch University	M21/11/025
Thailand	Chiang Mai University HIV Treatment (CMU HIV Treatment) CRS	Human Experimentation Committee, Research Institute for Health Sciences, Chiang Mai University	Study: 35/64<br>Initial certificate: 44/2022
		Research Ethics Committee Panel 3, Faculty of Medicine, Chiang Mai University	Study: NONE-2564-08666<br>Initial certificate: 177/2022
	Siriraj Hospital, Mahidol University NICHD CRS	Siriraj Institutional Review Board	Study: 962/2564(IRB2)<br>Initial certificate: Si 1005/2021
	Chiangrai Prachanukroh Hospital NICHD CRS	Central Research Ethics Committee (CREC)	Study: CREC093/64BP-PED9<br>Initial certificate: COA-CREC042/2022
		Ethics Committee in Human Research, Chiangrai Prachanukroh Hospital	Study: EC CRH 046/65 Ex<br>Initial certificate reference: CR 0033.102/EC 658
